# Comparative study of convolution, superposition, and fast superposition algorithms in conventional radiotherapy, three-dimensional conformal radiotherapy, and intensity modulated radiotherapy techniques for various sites, done on CMS XIO planning system

**DOI:** 10.4103/0971-6203.48716

**Published:** 2009

**Authors:** K. R. Muralidhar, Narayana P. Murthy, Alluri Krishnam Raju, NVNM Sresty

**Affiliations:** Department of Radiation Oncology, Indo-American Cancer Institute and Research Center, Road No:14, Banjara Hills, Hyderabad-500 034, Andhra Pradesh, ^1^Physics, Nagarjuna University, Nagarjuna Nagar, Guntur, Andhra Pradesh, India

**Keywords:** Algorithm, conformity index, homogenity index, treatment planning system

## Abstract

The aim of this study is to compare the dosimetry results that are obtained by using Convolution, Superposition and Fast Superposition algorithms in Conventional Radiotherapy, Three-Dimensional Conformal Radiotherapy (3D-CRT), and Intensity Modulated Radiotherapy (IMRT) for different sites, and to study the suitability of algorithms with respect to site and technique. For each of the Conventional, 3D-CRT, and IMRT techniques, four different sites, namely, Lung, Esophagus, Prostate, and Hypopharynx were analyzed. Treatment plans were created using 6MV Photon beam quality using the CMS XiO (Computerized Medical System, St.Louis, MO) treatment planning system. The maximum percentage of variation recorded between algorithms was 3.7% in case of Ca.Lung, for the IMRT Technique. Statistical analysis was performed by comparing the mean relative difference, Conformity Index, and Homogeneity Index for target structures. The fast superposition algorithm showed excellent results for lung and esophagus cases for all techniques. For the prostate, the superposition algorithm showed better results in all techniques. In the conventional case of the hypopharynx, the convolution algorithm was good. In case of Ca. Lung, Ca Prostate, Ca Esophagus, and Ca Hypopharynx, OARs got more doses with the superposition algorithm; this progressively decreased for fast superposition and convolution algorithms, respectively. According to this study the dosimetric results using different algorithms led to significant variation and therefore care had to be taken while evaluating treatment plans. The choice of a dose calculation algorithm may in certain cases even influence clinical results.

## Introduction

It is of paramount importance for the modern Conformal Radiotherapy technique to have accuracy in dose calculations in almost all relevant clinical situations. One of these situations is the treatment of lung tumors where irradiation has to be planned under challenging conditions, for dose calculation.[1] The accuracy of patient dose predictions has continuously improved by moving from the simple scatter in homogeneity corrections over pencil beam algorithms to point kernel-based Convolution/Superposition methods.[2]

The functionality and quality of any treatment planning system (TPS) depends on the type of Algorithm used in the different steps of planning process. An algorithm is defined as sequence of instructions that operate on a set of input data, transforming that information into a set of output results that are of interest to the user.[3]

In the present study Convolution, Superposition, and Fast superposition Algorithms were used for all plans. The purpose of the present study was to compare the results from three different algorithms, for four different sites, representing varied heterogeneity conditions and using Conventional, 3DCRT, and IMRT Techniques. This also allowed us to know the suitability of an algorithm for the respective diagnosis and treatment techniques.

Conformity Index, Homogeneity Index, Mean Dose, and Mean Relative Difference have been used to evaluate the external beam plans. Furthermore, Dose Volume Histograms (DVH) for different structures were obtained, to quantify the dose to the other OARs.

## Materials and Methods

### Patients and IMRT treatment planning

Four Cancer patients with diagnosis of Ca lung, Ca esophagus, Ca hypopharynx, and Ca prostate were selected for this study. Doses of 3200 cGy, 4000cGy, 7560 cGy, and 4500 cGy, were prescribed to the planning target volume of Lung, Esophagus, Hypopharynx, and Prostate cases, respectively. Planning Target Volume (PTV) was derived by using 5mm isotropic expansion of the Clinical Target Volume (CTV), which in turn was derived from the macroscopic Gross Tumor Volume (GTV).To deal with conflicting dose objectives during the optimization process of IMRT, plan PTV was made to exclude other OARs by 5mm.[4] (a) The spinal cord, right parotid, and left parotid in case of the hypopharynx, (b) spinal cord and heart in case of the lungs, (c) bladder and rectum in case of the prostate, and (d) left lung, right lung, and spinal cord in case of the esophagus, were delineated as OARs. Treatment planning objectives for the target and OARs were tabulated as shown in Table 1. For each patient Conventional, 3DCRT, and IMRT Plans were created with a photon beam of 6 MV quality, using Convolution, Superposition, and Fast Superposition algorithms. Commercially available CMS XiO (Computerized Medical Systems, USA) Planning system was used for planning purposes.

**Table 1:** Prescription for target and OARs in Ca.Lung, Ca.Prostate, Ca.Esophagus, and Ca.Hypopharynx cases in IMRT

*Lung*	*IMRT Prescription*		*Rank*	*Objective*	*Dose (cGy)*	*Volume (%)*	*Weight*	*Power*
		
	*Structure*	*Type*						
	PTV	Target	1	Maximum	3300	0	100	2
				Minimum	3200	100	300	2
	Heart	OAR	1	Maximum	1500	0	300	2
	Spinal Cord	OAR	2	Maximum	1500	0	100	2
Prostate								
	*Structure*	*Type*	*Rank*	*Objective*	*Dose (cGy)*	*Volume (%)*	*Weight*	*Power*
	PTV	Target	1	Maximum	4600	0	100	2
				Minimum	4500	100	100	2
	Bladder	OAR	2	Maximum	4500	0	200	2
					3083	29	200	2
					2591	49	200	2
					1736	68	200	2
	Rectum	OAR	3	Maximum	4500	0	300	2
					3083	29	300	2
					2591	49	300	2
					1658	69	300	2
Esophagus								
	*Structure*	*Type*	*Rank*	*Objective*	*Dose (cGy)*	*Volume (%)*	*Weight*	*Power*
	PTV	Target	1	Maximum	4100	0	100	3
				Minimum	4000	100	100	3
	Lt Lung	OAR	2	Maximum	1000	100	100	3
	Rt Lung	OAR	2	Maximum	1000	100	100	3
	SC	OAR	3	Maximum	4000	100	100	2
HypoPharynx								
	*Structure*	*Type*	*Rank*	*Objective*	*Dose (cGy)*	*Volume (%)*	*Weight*	*Power*
	PTV	Target	1	Maximum	7660	0	700	2
		Target	2	Minimum	7560	100	700	2
	L Parotid	OAR	1	Maximum	2100	0	100	2
	R Parotid	OAR	2	Maximum	2100	0	100	2

### Dose calculation algorithm

As mentioned, three different calculation algorithms were used to compute the dose for all plans that were generated during the study. The XiO's fast-Fourier transform (FFT) convolution algorithm and the superposition (Wiesmeyer an Miften)[5] algorithms are similar, in that, they both compute the dose by convolving the total energy released in the patient with Monte Carlo-generated energy deposition kernels, computed by Mackie *et al.*[6] Kernel is the dose matrix generated per unit TERMA at the interaction site. Total Energy Released per unit Mass (TERMA) is the product of the mass attenuation coefficient and the primary energy fluence.[7] The choice of dose calculation algorithms is an important consideration[8–10] when using “high-ended” planning methodologies and comparing one method with another.

#### Convolution algorithm:

The energy deposited kernels of Mackie *et al.*[5] must be interpolated from spherical to Cartesian coordinates on a common grid with the TERMA, to perform FFT convolution. Sampling and interpolation of kernels from spherical to Cartesian coordinates is complicated by steep kernel gradients. Adaptive quadrature techniques ensure that the correct energy at and near the interaction point is represented in the Cartesian coordinates.

Comparison of calculation results indicates that incorrect doses are obtained if the effect of scatter from the neighbors is omitted over a large enough region. It is important that patient data be represented over a 3D volume because the scatter calculated at a point is based on the 3D volume of the scattering medium.

The required volume over which scatter of kernel contributions must be included and the maximum volume used in the XiO system is about 30cm in the forward direction, 5cm in the backward direction, and twice the field size dimension laterally (essentially the contributions from all interaction points must be accumulated). Sharpe an Battista[11] report using these ranges and Mackie et al.[12,13] report the same required lateral range.

Including dose contributions over such a large area requires significant computation time. This computation time can be reduced by performing separate calculations; one with the primary kernel, for which the calculation is performed at a high resolution, but over a small region, the other with a scatter kernel, where calculation is performed at a lower resolution, but over a large area, as proposed by Mackie *et al.*[8,5] This approach is possible since the primary kernels have extremely large gradients close to the interaction point, but they make no contribution beyond a few centimeters from the interaction point, whereas, the scatter kernels have smaller gradients, but contribute the dose over a much larger range. The XiO system performs a separate high and low resolution FFT Calculation for the primary and scatter kernels, achieves a time saving of about 65% over performing a single Calculation at high resolution.

#### Superposition algorithm:

The XiO superposition dose deposition method is an adaptation of the “collapsed cone” dose calculation method.[9] As with FFT Convolution, all superposition calculations are done in beam coordinates, and the dose in the beam coordinates is interpolated to the user specified calculation volume. It is possible for superposition algorithms to directly emulate the kernel calculation process; that is, to calculate deposited energy by spreading energy released (TERMA) at the interaction points, to points in the volume of interest, according to the distribution implied by the kernel. This method is known as the “interaction point of view”.

Unlike the FFT convolution algorithms, the superposition algorithm energy deposition kernels can be modified to account for variations in electron density. The density scaling method, based on O'Connor's theorem (O'Connor 1957),[14] is used to distort the kernels by finding the average density along the straight-line path between the interaction site and dose deposition site. Density scaling is a good approximation for scattered photons, because the photons travel in straight lines and the mass attenuation coefficient scales linearly with the material density (assuming that the atomic number remains unchanged).

#### Fast superposition algorithm:

Spherical kernels, or “dose spread arrays”, are cylindrically symmetric and defined in terms of rays traced along zenith and azimuth angles. The spherical kernel computation has been augmented with the ability to combine (select and sum) adjacent zenith rays in the kernel. Thus, it is possible to limit the number and direction of zenith rays for the purpose of optimizing speed/accuracy tradeoffs: The more the rays, the slower and more accurate the calculation: the fewer the rays, the faster and less accurate the calculation. Control of both the direction and number of zenith rays and azimuth rays is possible, although the azimuth angles must be evenly spaced. The fast mode provides a fast superposition dose calculation with a speed-up factor of 2.5cm at the cost of a small loss in accuracy, compared to the “standard” superposition calculation.

### Dose reporting and evaluation

Table 1 shows the prescription for target and OARs in Ca.Lung, Ca.Prostate, Ca.Esophagus, and Ca.Hypopharynx cases in IMRT. The Rank, Weight, and Power that are mentioned in Table 1 are useful for better optimization and planning. Weight is an optimization used to increase the relative importance of a dose objective. Weight values range from 1 to 100. Weight can be used to increase or decrease the dose over the entire volume of the structure. Power is an option that can be used to increase the penalty to those voxels, with doses in violation of a structure's objective. The values of power range from 2.0 to 5.0, in increments of 0.1. A small increase in power can make a large change in the objective function of the curve. Ranks will give the preference in calculating the particular voxels.

For each patient, Dose - Volume histograms (DVHs) were generated using the CMS XiO Planning system for Conventional, 3DCRT, and IMRT plans [Figure 1a to Figure 1l]. Individual Dose-Volume points were also recorded. Dmin, Dmax, and Dmean were evaluated for the PTV and OARs. Conformity Index and Uniformity Index were calculated for PTV in all cases. Monitor Units were recorded in case of IMRT for all calculation algorithms. In all cases maximum variations of Dmin, Dmean, and Dmax were tabulated. Relative dose volume differences (percentage) between the results from the different dose calculation algorithms were computed for each diagnosis and technique. Maximum percent variations between algorithms were recorded for PTV, for all cases. All the sets of treatment plans were evaluated using a set of evaluation parameters, which complied with the evaluation criteria recommended by International Commission on Radiation Units and Measurements (ICRU) Report 62.[15,16] The evaluation parameters included the Conformity Index (CI) and the Homogeneity index (HI). The CI was defined as the quotient of the treated volume and the volume of the PTV.[16] The conventionally used homogeneity index (*H*-index) is defined as the ratio of the maximum dose in the PTV to the prescription dose,[17] with a value closer to 1 indicating better homogeneity. The *H*-index generally varies from 1 to 1.5 in the real-world patient treatment plans. The index's simplicity has led to its being extensively used for quantifying dose homogeneity in tumor volumes. For the evaluation of doses to the OARs, the mean dose was used. The plans that were done on the CMS planning system (with convolution, superposition, and fast superposition) were compared with the Direx, Accusoft planning system with the convolution algorithm, on the same patient CT data, and the results were tabulated.

**Figure 1a:**
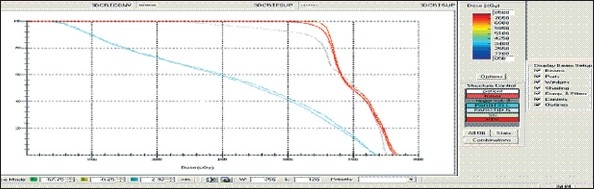
Hypopharynx 3DCRT - DVH with three algorithms

**Figure 1b:**
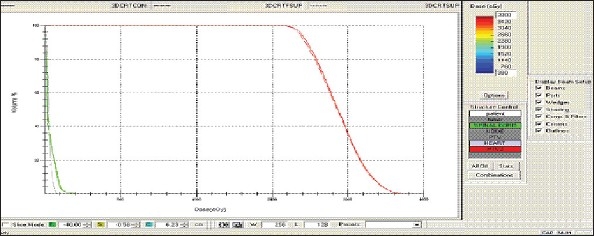
Lung - 3DCRT DVH with three algorithms

**Figure 1c:**
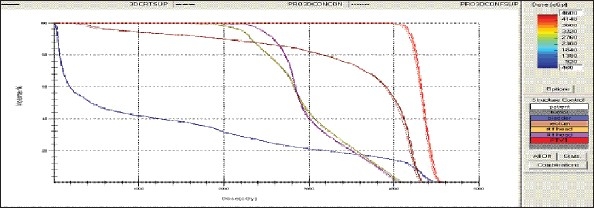
Prostate - 3DCRT - DVH with three algorithms

**Figure 1d:**
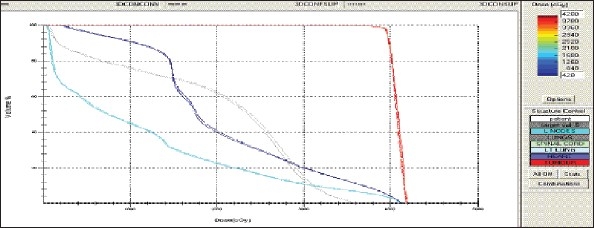
Esophagus - 3DCRT - DVH with three algorithms

**Figure 1e:**
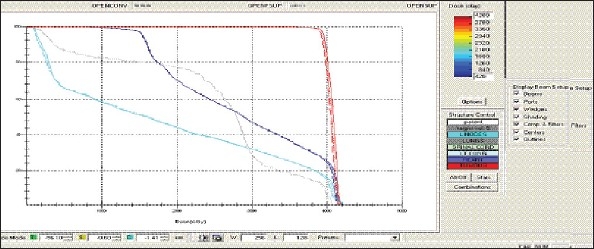
Esophagus - OPEN Technique - DVH with three algorithms

**Figure 1f:**
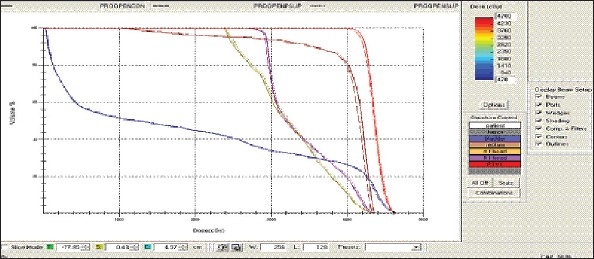
Prostate - OPEN Technique - DVH with three algorithms

**Figure 1g:**
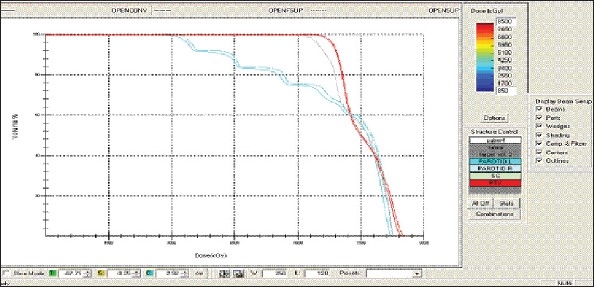
Hypopharynx - OPEN Technique - DVH with three algorithms

**Figure 1h:**
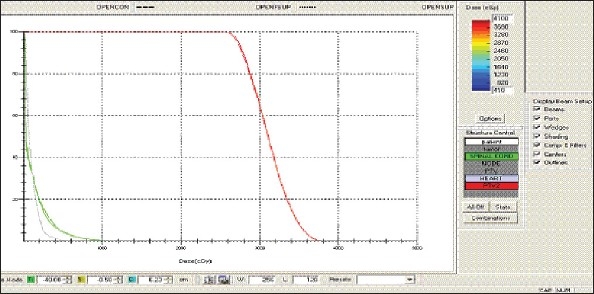
Lung - OPEN Technique - DVH with three algorithms

**Figure 1i:**
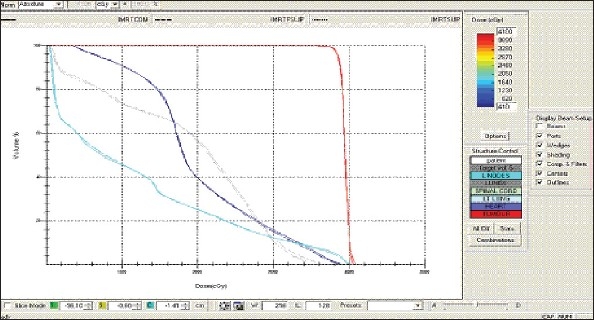
Esophagus - IMRT Technique - DVH with three algorithms

**Figure 1j:**
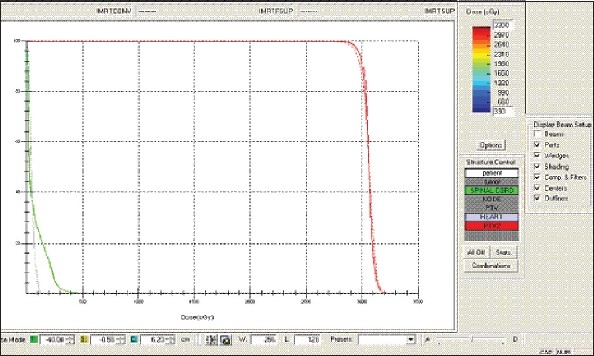
Lung - IMRT Technique - DVH with three algorithms

**Figure 1k:**
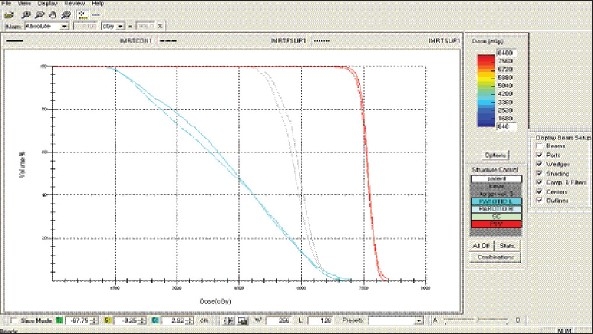
Hypopharynx – IMRT Technique DVH with three algorithms

**Figure 1l:**
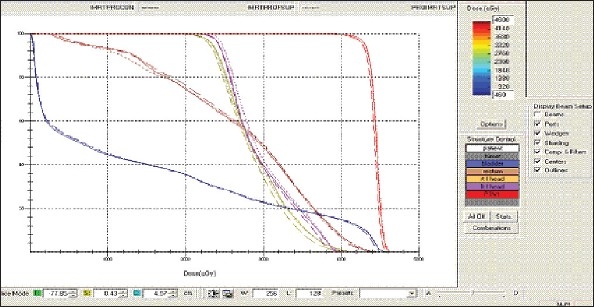
Prostate - IMRT Technique DVH with three algorithms

## Results

Table 2 and 3 show the results of the statistical analysis. Mean Relative difference from the prescribed doses for the target are reported in Table 2, for all diagnoses and treatment techniques. Fast Superposition algorithm shows the minimum deviation from the prescription dose in the lung and esophagus cases, for all techniques. The Convolution algorithm shows good results in case of the Hypopharynx for all techniques. In case of the prostate, the Superposition algorithm shows better in the conventional technique and the Fast superposition algorithm in the 3DCRT and IMRT techniques. Summary of results are reported in Table 3.

**Table 2:** Mean relative differences with prescribed dose with three algorithms

*Technique*	*Organ*	*Mean relative difference (%)*			Var*
			
		*Convolution*	*Superposition*	*Fast superposition*	
Conventional	Prostate	−0.32	−0.2	−0.64	0.44
3DCRT	Prostate	−0.71	−1.3	0.59	0.59
IMRT	Prostate	1.37	1.39	0.8	0.59
Conventional	Hypopharynx	2.89	3.36	3.31	0.47
3DCRT	Hypopharynx	2.34	2.68	2.55	0.34
IMRT	Hypopharynx	−0.38	−0.91	−1.1	0.72
Conventional	Esophagus	2.47	1.91	1.61	0.86
3DCRT	Esophagus	2.8	2.62	2.42	0.38
IMRT	Esophagus	0.96	0.58	0.4	0.56
Conventional	Lung	1.3	1.47	1.17	0.3
3DCRT	Lung	0.91	0.91	0.55	0.36
IMRT	Lung	−0.61	−0.55	−0.49	0.12

* Var: Percentage of variation between algorithms in mean relative difference

**Table 3:** Summary of convolution, superposition, and fast superposition algorithms in different sites and in different treatment techniques

		*Hypopharynx*			*Lung*			*Prostate*			*Esophagus*		
					
		*Conventional*	*3DCRT*	*IMRT*	*Conventional*	*3DCRT*	*IMRT*	*Conventional*	*3DCRT*	*IMRT*	*Conventional*	*3DCRT*	*IMRT*
PTV	Maximum % of difference in Dmin	0.33	1.7	0.48	0.7	0.7	3.7	0.8	0.9	0.5	0.4	1.04	2.9
	Maximum % of difference in Dmax	0.99	0.8	2.2	0.4	0.7	0.7	0.3	0.5	1.2	0.9	0.5	1.5
	Maximum % of difference in Dmean	0.5	0.3	0.2	0.3	0.3	0.13	0.4	0.6	0.6	0.8	0.4	0.05
	Dmin is in	CON	CON	CON	FSUP	FSUP	SUP	FSUP	FSUP	CON	FSUP	CON	CON
	Dmax is in	FSUP	FSUP	FSUP	SUP	SUP	SUP	CON	CON	SUP	CON	CON	FSUP
	Nearest to mean dose is with	CON	CON	CON	FSUP	FSUP	FSUP	SUP	CON	SUP	FSUP	FSUP	FSUP
OAR1		*Spinal cord*			*Spinal cord*			*Bladder*			*Left Lung*		
					
	Maximum dose of OAR1 is in	SUP	SUP	CON	SUP	FSUP	CON	CON	CON	CON	CON	CON	CON
	Minimum dose of OAR1 is in	CON	CON	CON	NONE	NONE	NONE	SUP	SUP	SUP	CON	FSUP	SUP
	Maximum % of difference in Dmean	0.12	0.1	1.8	1.92	0	1.6	0.6	0.36	0.7	0.3	0.9	1.35
OAR2		*Rt parotid*			*Heart*			*Rectum*			*Spinal cord*		
					
	Maximum dose of OAR2 is in	SUP	SUP	CON	FSUP	FSUP	FSUP	SUP	CON	SUP	CON	SUP	CON
	Minimum dose of OAR2 in	CON	CON	CON	FSUP	FSUP	FSUP	CON	CON	CON	FSUP	FSUP	FSUP
	Maximum % of difference in Dmean	1.7	1.87	0.94	3.3	11	10	0.7	0.4	1.27	0.4	1.5	0.8
OAR3		*Lt parotid*									*Right Lung*		
				
	Maximum dose of OAR3 is in	SUP	SUP	CON							CON	CON	CON
	Minimum dose of OAR3 is in	CON	CON	CON							CON	CON	CON
	Maximum % of difference in Dmean	1.8	1.8	2							1.5	0.86	0.9

Con: Convolution Algorithm, Sup: Superposition Algorithm, Fsup: Fast Superposition Algorithm, Dmin: Minimum Dose, Dmax: Maximum Dose, Dmean: Mean dose, Minimum Relative differences with prescribed doses to PTV are shown in Table 4. For three algorithms; the mean relative differences in dose volume value with the prescribed dose are also presented. Significant differences between three dose calculation algorithms can be observed in the result

Minimum Relative differences with prescribed doses to PTV are shown in Table 4. For three algorithms; the mean relative differences in dose volume value with the prescribed dose are also presented. Significant differences between three dose calculation algorithms can be observed in the result.

**Table 4:** Minimum relative difference with prescribed dose in four different sites and in different treatment techniques and algorithm

	*Technique*	*Algorithm*
Prostate	Conventional	SUPERPOSITION
	3DCRT	CONVOLUTION
	IMRT	SUPERPOSITION
Lung	Conventional	FAST SUPERPOSITION
	3DCRT	FAST SUPERPOSITION
	IMRT	FAST SUPERPOSITION
Esophagus	Conventional	FAST SUPERPOSITION
	3DCRT	FAST SUPERPOSITION
	IMRT	FAST SUPERPOSITION
Hypopharynx	Conventional	CONVOLUTION
	3DCRT	CONVOLUTION
	IMRT	SUPERPOSITION

1. Hypopharynx: When the mean relative differences are compared with the prescribed dose in the three algorithms, the convolution algorithm certainly shows the best correspondence within the target structures (within 0.5% for all dose volume points), for all techniques, and it also reports significantly lower doses to OARs. Maximum percentage variations for different algorithms for Dmin, Dmax, and Dmean are 1.7 2.2, and 0.5%, respectively for PTV. Maximum percentage variation for different algorithms for OAR's Dmean is observed to be 1.8% for spinal cord in IMRT, 1.87% for Right Parotid in 3DCRT, and 2.1% for Left Parotid in IMRT.
2. Lung: When the mean relative differences are compared with the prescribed dose in the three algorithms, the fast superposition algorithm certainly shows the best correspondence within the target structures (within 0.4% for all dose volume points), for all techniques, and it also reports significantly lower does to OARs. Maximum percentage variations in Dmin, Dmax, and Dmean are 3.7, 0.7 and 0.3% for PTV, when the dose calculation uses different algorithms. Maximum percentage variation for different algorithms in case of OAR's Dmean is observed to be 1.92% for Spinal cord for Conventional treatment and 11% for Heart in 3DCRT.
3. Prostate: When comparing the prescribed dose with the three algorithms, the superposition algorithm certainly shows the best correspondence within the target structures (within 1% for all dose volume points), for all techniques, although, the superposition algorithm reports significantly higher Dmax values to OARs. Maximum percentage variations for different algorithms in case of Dmin, Dmax and Dmean are 0.9, 1.2 and 2.1% for PTV. Maximum percentage variation in OAR's Dmean is observed to be 6.9% for Bladder in 3DCRT and 1.27% for rectum in 3DCRT, when evaluated using different algorithms.
4. Esophagus: When comparing the mean relative differences with the prescribed dose in the three algorithms, the fast superposition algorithm certainly shows the best correspondence within the target structures (within 1% for all dose volume points), for all techniques, and it also reports significantly lower doses to the spinal cord. Higher doses of OARs are due to the convolution technique.
  Maximum percentage of variation between algorithms in Dmin, Dmax, and Dmean are 2.9, 1.5, and 0.83% for PTV. The maximum percentage of variation observed between algorithms in OAR's Dmean is 1.35% for Left lung in IMRT, 1.5% for Spinal cord in 3DCRT, and 1.5% for Right Lung in Open treatment.
5. Homogeneity Index: Maximum Percentage variations between algorithms for Homogeneity Index values are 0.68,1.25,1.54, and 2.2% for Lung, Prostate, Esophagus, and Hypopharynx cases. Homogeneity Index values are presented in Table 5.
6. Conformity Index: Maximum Percentage variations between algorithms for Conformity Index values are 1.49, 2.2, 0.86, and 1.85% for Lung, Prostate, Esophagus and Hypopharynx cases, respectively. Conformity Index values are presented in Table 6.

**Table 5:** Homogeneity index

	*Algorithm*	*Homogeneity index*	*Max.% of variation*
Lung	CON	1.019	
	SUP	1.021	0.68
	FSUP	1.014	
Prostate	CON	1.031	
	SUP	1.038	1.25
	FSUP	1.025	
Esophagus	CON	1.021	
	SUP	1.034	1.54
	FSUP	1.037	
Hypopharynx	CON	1.084	
	SUP	1.108	2.2
	FSUP	1.109	

**Table 6:** Conformity index

	*Algorithm*	*Conformity index*	*Max.% of variation*
Hypopharynx	CON	1.08	
	SUP	1.06	1.85
	FSUP	1.06	
Prostate	CON	1.77	
	SUP	1.81	2.2
	FSUP	1.81	
Lung	CON	1.32	
	SUP	1.34	1.49
	FSUP	1.34	
Esophagus	CON	1.15	
	SUP	1.16	0.86
	FSUP	1.16	

The Number of Monitor Units was almost the same for all the algorithms that are shown in Figure 2a to Figure 2d. The difference in dose distribution between fast superposition and superposition algorithms in the prostate case with IMRT Technique is shown in Figure 3, in the form of a dose wash. The yellow color shows the deviation of 3%. The dose wash for convolution, superposition, and fast superposition algorithms for Lung, Prostate, Esophagus, and Hypopharynx cases are shown in Figure 4a to Figure 4d.

**Figure 2a:**
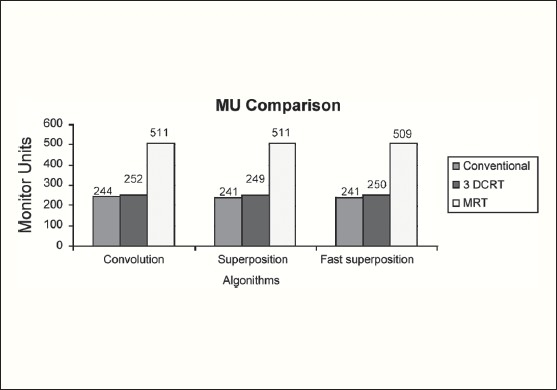
Monitor Unit comparison in Carcinoma of Esophagus

**Figure 2b:**
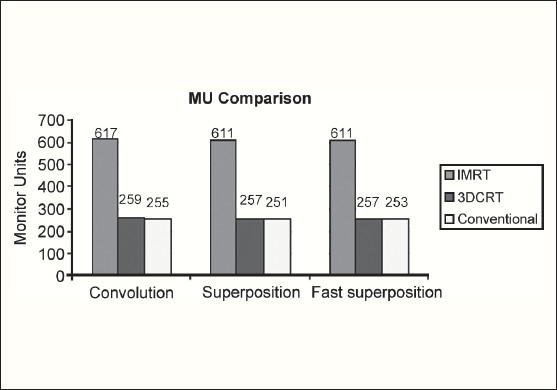
Monitor Unit comparison in Carcinoma of Prostate

**Figure 2c:**
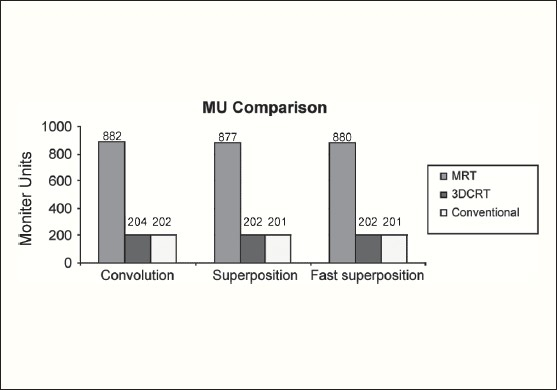
Monitor Unit comparison in Carcinoma of Hypopharynx

**Figure 2d:**
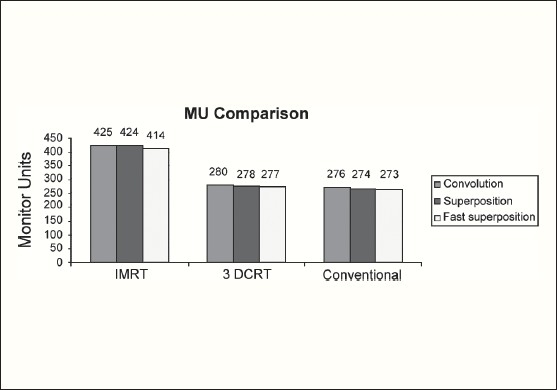
Monitor Unit comparison in Carcinoma of Lung

**Figure 3:**
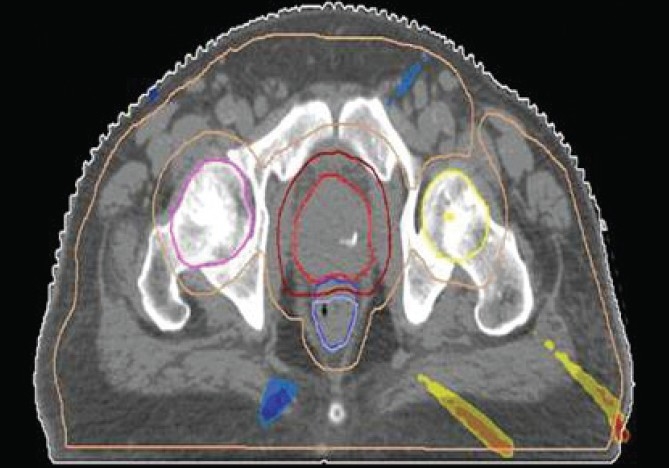
The difference in dose wash between Fast Superposition algorithm and Superposition algorithm in Ca. Prostate with IMRT technique

**Figure 4a:**
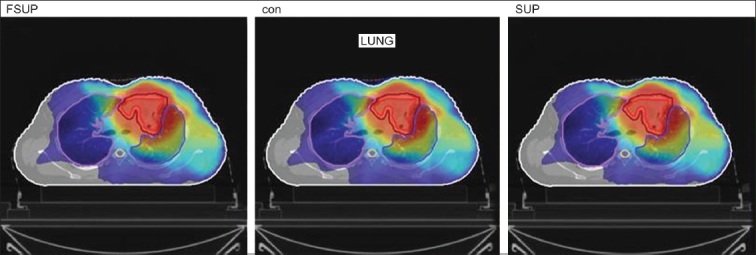
Dose wash with convolution, superposition, and fast superposition algorithms in ca.hypopharynx

**Figure 4b:**
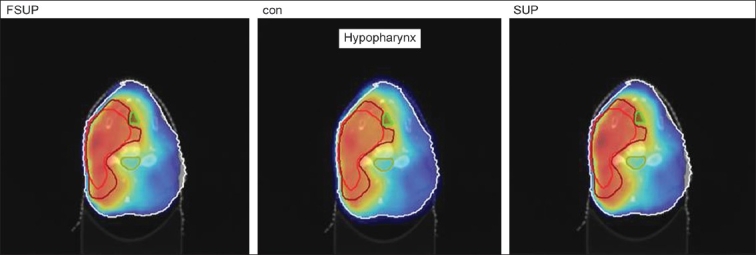
Doses wash with convolution, superposition, and fast superposition algorithms in ca.lung

**Figure 4c:**
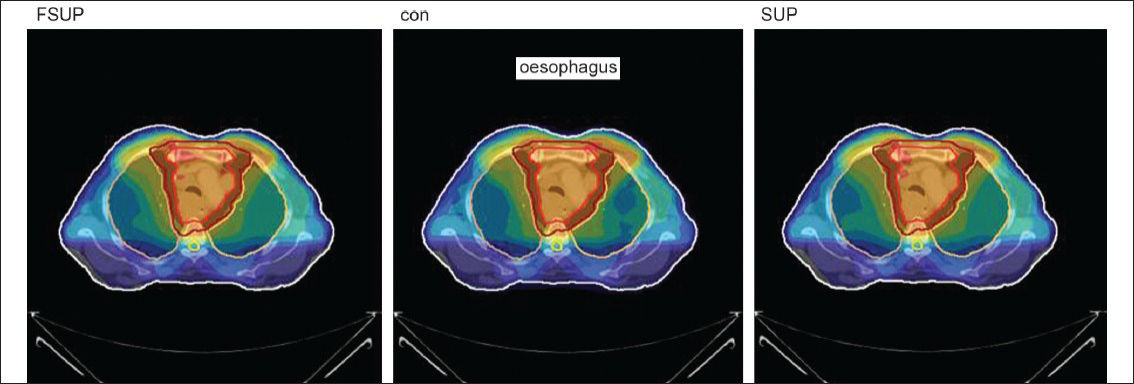
Dose wash with convolution, superposition, and fast superposition algorithms in ca.esophagus

**Figure 4d:**
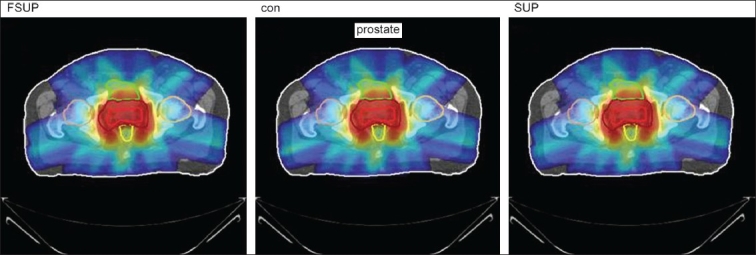
Doses wash with convolution, superposition, and fast superposition algorithms in ca.prostate

To illustrate the observed differences for individual patients Figure 1a to Figure 1l show an exemplary set of DVHs (for four diagnoses) of the PTV, and the OARs for all three dose calculation algorithms. Table 7 shows the Summary of Suitability of Algorithms with respect to diagnosis and treatment techniques. Table 8 shows the comparison between the CMS XIO and Direx Accusoft planning systems. The maximum percent of variation observed with respect to the Accusoft planning system was 0.9%, which proved that the CMS XIO planning system with all the three algorithms was suitable to start with, for clinical use.

**Table 7:** Summary of suitability of algorithms with respect to site and technique

*Diagnosis*	*Min. relative difference with prescribed dose*			*CI*	*HI*
			
	*Conventional*	*3DCRT*	*IMRT*		
Ca.Lung	FSUP	FSUP	FSUP	CON	FSUP
Ca.Esophagus	FSUP	FSUP	FSUP	CON	CON
Ca.Hypopharynx	CON	CON	CON	SUP	CON
Ca.Prostate	SUP	CON	SUP	CON	FSUP

CI = Conformity Index, HI = Homogeneity Index, Min = Minimum, Ca. = Carcinoma

**Table 8:** Tumor dose comparison between CMS XIO planning system and direx accusoft planning system

*Planning system*	*Algorithm*	*Mean dose (cGy)*	*Min dose (cGy)*	*Max dose (cGy)*
Direx, Accusoft	Convolution	4920	4750	5025
CMS, XIO	Convolution	4927	4705	5016
	Superposition	4943	4703	5016
	Fast Superposition	4944	4708	5018
Max % of variation between two planning systems		−0.48	0.92	0.17

## Discussion

The XiO's fast-Fourier transform (FFT) convolution algorithm and the superposition algorithms are similar, in that, they both compute the dose by convolving the total energy released in the patient, with Monte Carlo-generated energy deposition kernels, computed by Mackie et al. Unlike the FFT convolution algorithms, the superposition algorithm energy deposition kernels can be modified to account for variations in electron density. The fast superposition dose calculation algorithm is 2.5 times faster than the superposition algorithm with small loss in accuracy. Significant deviations are observed between these three dose calculations algorithms. Therefore, substantial errors can be made when an insufficiently accurate dose computation algorithm is selected. Comparison with measurements has shown that the Fast superposition algorithm performs excellently for Lung cases. For treatment planning of lung cancer, it is highly important to take into account differences in tissue density during dose computation and to model the secondary electron transport accurately. From Tables 4 and 7 it can be seen that not a single algorithm is close to prescription for any diagnosis in our study. Fast superposition algorithm performs excellently in Lung and Esophagus cases. These two sites are more inhomogeneous compared to other sites taken for comparison. These results are shown in Tables 4 and 7. This algorithm shows good results for all three techniques (conventional, 3DCRT, and IMRT). Similarly the superposition algorithm is good in the prostate case and convolution algorithm proves to be good in the hypopharynx case. The DVHs of the different dose calculation algorithms clearly show the deviations between them. The DVHs are also shown for each diagnosis and technique. The deviations in IMRT are to be concentrated more upon because of the usage of small segments. To evaluate the performance of the inhomogeneity correction, 3DCRT plans are more crucial, because of the smaller number of treatment fields. On the other hand, the fact that IMRT beam segments will generally be smaller than the 3D field shapes, it will potentially increase dose errors for IMRT. In IMRT cases the larger errors are due to MLC Leakage and Tongue and Groove effect.

## Conclusion

We compared Convolusion, Superposition, and Fast superposition algorithms (CMS, XiO Planning system) using Conventional, 3DCRT, and IMRT techniques for Esophagus, Lung, Prostate, and Hypopharynx cases. Within the target structures the deviations of mean dose to the prescribed dose and maximum percentage of variation between all algorithms were recorded for all techniques. Maximum percentage of variation between algorithms was 3.7%, recorded in case of Ca Lung with IMRT technique. Statistical analyses were performed by comparison of mean relative differences with prescribed dose, and Conformity Index and Homogeneity Index for target structures, and are shown in Table 7. This planning system was compared with Direx Accusoft planning system for clinical validation. The fast superposition algorithm showed excellent results for lung and esophagus cases by considering the mean relative differences with prescribed dose with three algorithms from Table 2 and minimum relative difference with prescribed dose in four different sites and in different treatment techniques from Table 4. These tables also show that the superposition algorithm is excellent for prostate, for all techniques, and the convolution algorithm is good for the hypopharynx cases, in conventional cases. The major differences are that convolution does not calculate dose as accurately as superposition in the presence of tissue inhomogeneities. In cases of cancer of lung, prostate, esophagus, and hypopharynx, organs at risk are getting more doses with superposition algorithm, convolution algorithm, fast superposition algorithm, and convolution algorithms, respectively. According to this study as the results from these algorithms differed, significant care should be taken when evaluating treatment plans, as the choice of the dose calculation algorithm may influence treatment planning as well as clinical results.
